# Two-Dimensional Borophene: Properties, Fabrication, and Promising Applications

**DOI:** 10.34133/2020/2624617

**Published:** 2020-06-15

**Authors:** Zhongjian Xie, Xiangying Meng, Xiangnan Li, Weiyuan Liang, Weichun Huang, Keqiang Chen, Jianming Chen, Chenyang Xing, Meng Qiu, Bin Zhang, Guohui Nie, Ni Xie, Xiaobing Yan, Han Zhang

**Affiliations:** ^1^Key Laboratory of Optoelectronic Devices and Systems of Ministry of Education and Guangdong Province, Institute of Microscale Optoelectronics and Otolaryngology Department and Biobank of the First Affiliated Hospital, Shenzhen Second People's Hospital, Health Science Center, Shenzhen University, Shenzhen 518060, China; ^2^Shenzhen International Institute for Biomedical Research, 518116 Shenzhen, Guangdong, China; ^3^National-Local Joint Engineering Laboratory of New Energy Photovoltaic Devices, Key Laboratory of Digital Medical Engineering of Hebei Province, College of Electron and Information Engineering, Hebei University, Baoding 071002, China; ^4^Nantong Key Lab of Intelligent and New Energy Materials, College of Chemistry and Chemical Engineering, Nantong University, Nantong, 226019 Jiangsu, China; ^5^Center for Stretchable Electronics and Nanoscale Systems, Key Laboratory of Optoelectronic Devices and Systems of Ministry of Education, College of Physics and Optoelectronic Engineering, Shenzhen University, Shenzhen 518060, China; ^6^Key Laboratory of Marine Chemistry Theory and Technology (Ocean University of China), Ministry of Education, Qingdao 266100, China

## Abstract

Monoelemental two-dimensional (2D) materials (Xenes) aroused a tremendous attention in 2D science owing to their unique properties and extensive applications. Borophene, one emerging and typical Xene, has been regarded as a promising agent for energy, sensor, and biomedical applications. However, the production of borophene is still a challenge because bulk boron has rather intricate spatial structures and multiple chemical properties. In this review, we describe its excellent properties including the optical, electronic, metallic, semiconducting, photoacoustic, and photothermal properties. The fabrication methods of borophene are also presented including the bottom-up fabrication and the top-down fabrication. In the end, the challenges of borophene in the latest applications are presented and perspectives are discussed.

## 1. Introduction

Graphene is the first discovered 2D material [1]. The discovery of the astonishing properties of graphene has brought forth a series of new materials known as “2D materials” [2–4]. 2D forms are a comparatively exciting and new area for many applications [5, 6]. Usually, 2D materials have many prominent physical properties that are promising for electronic devices, nanoengineering, energy conversion, and photonics [7–11]. With the rapid development of graphene, 2D materials, such as phosphorene, BN, germanene, antimonene, silicene, arsenene, and transition metal dichalcogenides, have recently arisen extensive interest. A mass of atom-thick materials have been theoretically predicted or synthesized [12–16]. More surprisingly, they possess different structures from graphene, owing to the different out-of-plane buckling degrees [17]. Besides the 2D materials exfoliated from their layered bulk counterparts, some 2D materials can also be manufactured from the bulk materials without layered form, such as the compound of 2D flat boron, 2D GaN, and hafenene [18, 19].

The research on boron in various compounds can be traced back to several hundred years ago, because boron possesses the extraordinary property to combine with nearly all of the other elements. Among them, hexagonal boron nitride (h-BN) is a wide bandgap III-V compound. It is a layered material with a graphite-like structure in which planar networks of h-BN hexagons are regularly stacked. h-BN possesses a high chemical stability, excellent physical properties, and a high thermal conductivity [20–23]. It is very similar to graphite, so that one may expect to prepare pure boron. In Sands' work, the pure boron was first documented in 1957 and the bulk g-B106 with an extremely complicated structure was reported. Up to now, bulk boron is widely known to have more than 16 polymorphs, all featuring interlinked polyhedra but only a few having identified crystal structure [17].

Since boron locates between nonmetallic carbon and metallic beryllium, there are merely three valence electrons in boron: [He]2s^2^2p^1^. The 2p electron and its orbit radius are near the 2s state, endowing it both metallicity and nonmetallicity. In bulk boron, the particular electronic structure empowers the formation of greatly diverse bonding and facilitates the extraordinary bond formation. 2D boron provides greater energy relief, compared to any other 2D materials [17, 24].

In 2015, the 2D boron sheet was successfully fabricated on argentum (Ag) substrates [25]. The study of borophene has attracted a lot of researchers in many fields, such as material science, nanotechnology, physics, chemistry, and condensed matter [13, 26, 27]. “Borophene” is the potential new atom-thick boron nanosheets for the large-scale synthesis [28]. It is the lightest 2D material to date. Borophene is the neighbor of graphene, and thus, it is desired to possess some similar properties to graphene [29]. Both *σ* and *π* electrons in borophene occupy the electronic states of the Fermi surface, making it superconductive. There are no high pressure and external strain; borophene could have the highest *T*_*c*_ among the 2D materials. For 2D boron structures, the chemical and structural complexity, electronic properties, and stability have been studied extensively [27, 30, 31].

The mechanical properties of borophene are particularly interesting and important. Firstly, borophene has low mass density. Provided that its ideal strength and in-plane stiffness are satisfactorily high, borophene can be used as assist elements for designing composites. Secondly, borophene is suitable for fabricating flexible nanodevices because of the high standards of flexibility against off-plane deformation [32–34]. Moreover, because of the powerfully anisotropic structure in borophene, its magnetic and electronic properties can be effectively controlled for multiple applications [35–37]. As the boron atoms are rich in bonding configurations, borophene is polymorphic, further differentiating it from other 2D materials [38, 39]. The low mass density of boron also results in the strong electron-phonon coupling, within the scope of 10-20 K which causes phonon-mediated superconductivity with high critical temperature [40, 41]. In a word, borophene is rich in resources, has low atomic weight, is lightweight, is low cost, and has excellent electrical performances. These advantages of borophene provide it more possibilities for practical application in the future.

Although borophene has many potential applications, the synthesis and discovery of its neoatomic structures with well-designed structure-property relationships retain among the most severe challenges. In extra, for synthetic 2D materials, the resulting atomic structure is influenced by multiple factors, such as the constituent elements, processing conditions, and growth substrates [7]. In order to realize practical applications, insured synthesis of quality specimens and the separation of borophenes from substrates remain challenging, requiring continuing experimental and theoretical efforts [17].

**Figure 1:**
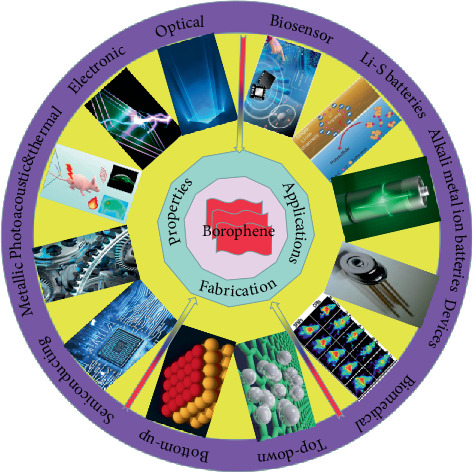
Comprehensive overview diagram of borophene.

In this review, we introduced the different experimental fabrication methods, the physicochemical properties, and the latest applications of borophene (Figure 1). The experimental synthesis includes bottom-up fabrication and top-down fabrication. The physicochemical properties of borophene mainly contain the optical, electronic, semiconducting, photoacoustic and photothermal, and metallic properties. Finally, we summarized the application of borophene in many fields, such as Li-S batteries, alkali metal ion batteries, and sensor and biomedical applications.

## 2. Theory and Properties

### 2.1. Optical Properties

The complex dielectric function of borophene benzene is *ε*(*ω*) = *ε*_1_(*ω*) + *iε*_2_(*ω*), which determines its optical properties. In the case of metals, the sum of interband and intraband components constitutes the dielectric tensor. We do not take into account factors other than visible regions and interband transitions, so the dielectric function in druid region (low frequency) (1), (2) may not be accurate. The imaginary part *ε*_1_^*αβ*^(*ω*) = 1 + (2/*π*)*P*∫_0_^∞^(*ε*_2_^*αβ*^(*ω*′)*ω*′/*ω*′^2^ − *ω*^2^ + *iη*)*dω*′, (2) of the mediation tensor is confirmed by summing the empty band parts using (3).

(1) $$\begin{array}{c} \varepsilon_{2}^{\alpha \beta }\left(\omega \right)=\frac{2\pi e^{2}}{\Omega \varepsilon_{0}}\underset{k,v,c}{\sum }\delta \left(E_{k}^{c}-E_{k}^{v}-h\omega \right){\left|\langle \Psi_{k}^{c}\left|u\cdot r\right|\Psi_{k}^{v}\rangle \right|}^{2}. \end{array}$$

*ε*_0_ is the dielectric constant of the vacuum, *ω* is for volume, *v* and *c* explicit the *E*_*v*_ and *E*_*c*_, *ω* express the energy of the object phonon, *u* · *r* stands for momentum symbol, and Ψ_*k*_^*c*^ and Ψ_*k*_^*v*^ express the conduction band and valence band at point *K*. The real part *ε*_1_^*αβ*^(*ω*) of the dielectric tensor is obtained from the Kramers-Cronin relation:

(2) $$\begin{array}{c} \varepsilon_{1}^{\alpha \beta }\left(\omega \right)=1+\frac{2}{\pi }P\int_{0}^{\infty }\frac{\varepsilon_{2}^{\alpha \beta }\left(\omega^{\prime }\right)\omega^{\prime }}{\omega \prime^{2}-\omega^{2}+i\eta }d\omega^{\prime }. \end{array}$$

The absorption constant *α*(*ω*) and *R*(*ω*) can be calculated from functions (4) and (5) according to the above-mentioned optical characteristics of borophene such as dielectric function and energy loss spectrum *L*(*ω*).

(3) Lω=Im−1εω=ε2ωε12ω+ε22ω,

(4) $$\begin{array}{c} \alpha \left(\omega \right)=\frac{\sqrt{2}\omega }{c}{\left\{\left[\varepsilon_{1}^{2}\left(\omega \right)+\varepsilon_{2}^{2}\left(\omega \right)\right]\right.}^{1/2}-{\left.\varepsilon_{1}\left(\omega \right)\right\}}^{1/2}, \end{array}$$

(5) $$\begin{array}{c} R\left(\omega \right)={\left|\frac{\sqrt{\varepsilon_{1}\left(\omega \right)+i\varepsilon_{2}\left(\omega \right)}-1}{\sqrt{\varepsilon_{1}\left(\omega \right)+i\varepsilon \left(\omega \right)}+1}\right|}^{2}. \end{array}$$

By calculating the dielectric equation and the electron energy loss equation of the incident radiation after the polarity of the electric field vector *E* in the directions of a and b, it can be seen that the crystal structure of borophene is anisotropic, resulting in the anisotropy in its optical properties.

**Figure 2:**
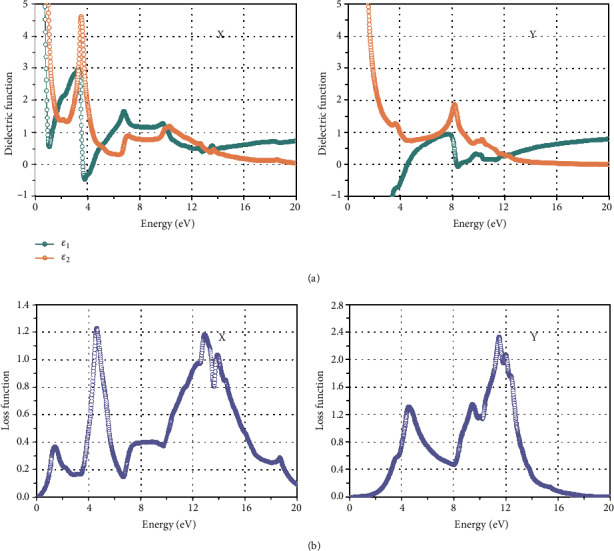
The optical response of polarized light on the *X* and *Y* axes to the illumination of 2D borophene cells, as illustrated in (a) and (b). Reprinted with permission from Ref. [35]. Copyright 2016 Royal Society of Chemistry.

In Figure 2(a), the virtual section of the dielectric medium model function quickly decreases at small frequencies close to the area of free electron. Direction of light polarization *X*, *ε*_2_(*ω*), increases fast at 2.41 eV and then up to the peak at 3.52 eV. Interband transitions from many occupied *K* states under electromotive force appear at the consistent energy, resulting in a powerful spike for direction of light polarization *Y* and the peak *ε*_2_(*ω*) is stronger than other peaks at 8.14 eV.

Due to the anisotropic crystal structure of borophene, there is a huge in-plane anisotropy discovered in the optical properties [13]. Borophene is a material showing promising potential in photovoltaic, flexible electronics, and display technologies, on account of the high electrical conductivity and optical transparency along the a direction, and all kinds of novel anisotropy [35, 42, 43].

Tai et al. have measured the optical bandgap of boron, which was 2.25 eV. It is approaching the value affirmed by first-principles calculations, which was 2.07 eV. They observed through strong photoluminescence and found that the borophene is a charming direct bandgap semiconductor [44]. Their ability to emit and absorb light of semiconductors is directly influenced by the electronic band structures. Wang et al. found that, the direct bandgap semiconductors, photons with energy surpass the bandgap energy which could be easily emitted or absorbed. For indirect bandgaps, there must be an additional phonon emitted or absorbed to make up for the energy imbalance, making the efficiency of photon emission or absorption process decreased [5].

**Figure 3:**
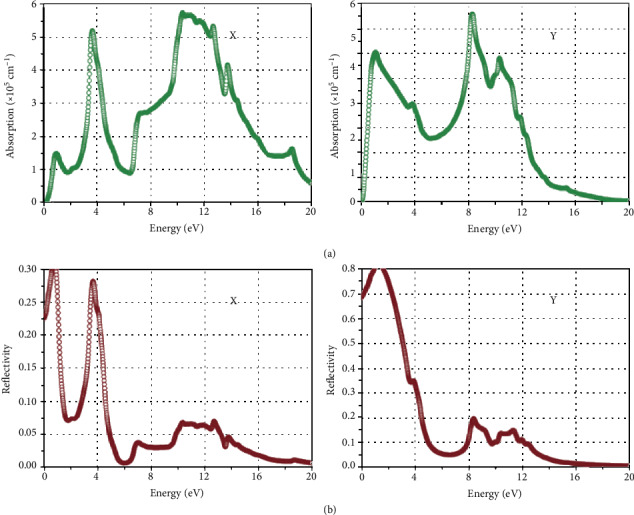
(a) Absorption coefficient of borophene and (b) reflectivity alongside the different two directions. Reprinted with permission from Ref. [35]. Copyright 2016 Royal Society of Chemistry.

Figure 3 reveals the absorption coefficient and reflectivity. In the two plane directions, the main absorptions are in the different positions. Along a direction, the major absorption peaks are situated at about 10.36 and 3.65 eV. Along b direction, the absorption regions can be up to 10.31, 8.29, and 1.09 eV. Moreover, the reflectivity of borophene along the two directions is lesser than 30% and higher than 40%, respectively, in the visible region. Along the uncorrugated a direction, the transmittance of borophene is high, and the electrical conductivity is very high. However, in the visible area, the optical conductivity of borophene is very small. Such properties offer chances for applications in flexible electronics, photovoltaic, and display technologies [35].

Adamska et al. find that borophene is an anisotropic metal, with a faint absorbance in the visible area, thickness-dependent optical transparency, and strong energy [45]. Tatullo et al. demonstrate that in the visible range, borophene is a weak absorber, which holds high optical transparency, resulting in excellent transparency [46]. Moreover, Lherbier et al. show a strong photosensitivity of borophene as regards any surface modifications [47].

### 2.2. Electronic Properties

The crystal structure of borophene is highly anisotropic [35]. Lots of researches foretell that different low-energy crystal structures may lead to metallic or semimetallic behavior. They may include Dirac cones near or at the Fermi energy and show anisotropy of conductivity because of their anisotropic bonding configuration [45, 48]. 2D boron exhibits diversiform structural polymorphs. Although the 2D and 3D forms of boron are alike in the organizations of chemical bonds, every 2D boron polymorph is metallic but the nature of their 3D forms is diverse [17].

**Figure 4:**
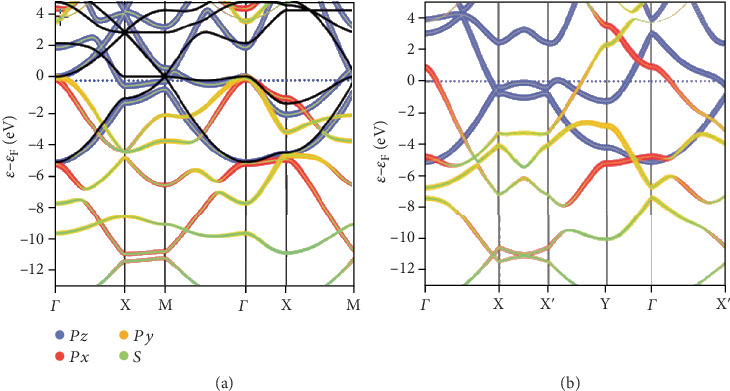
Electronic properties of 2D boron. The local density approximation band structures of the (a) *v*1/6 and (b) *v*1/5 sheets in a vacuum. Reprinted with permission from Ref. [49]. Copyright 2016 American Chemical Society.

The band structures of the *v*1/6 and *v*1/5 2D boron sheets computed employing the LDA are shown in Figures 4(a) and 4(b). Most of the states are metallic root in the 2pz state, around the Fermi level, which is greatly delocalized above a huge energy window. Under circumstance of the *v*1/5 2D boron sheet, the metallicity is given by the 2px and 2py states, which will generate a bandgap close to the Fermi level in the 2D boron sheets with the value of *v* between 10% and 15%. The estimated metallicity was confirmed by the latest experimental methods [17].

**Figure 5:**
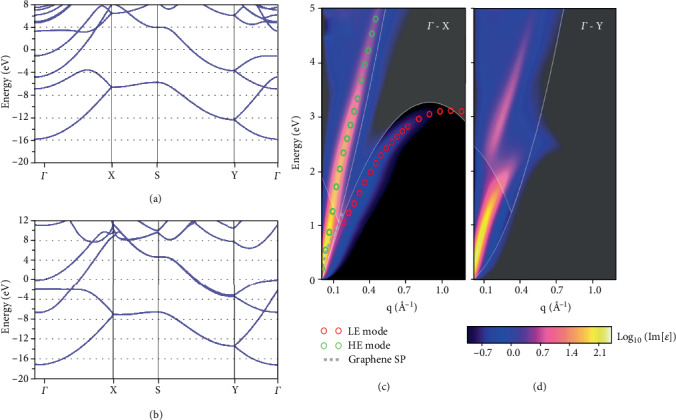
The electron band structure of borophene through (a) PBE and (b) HSE06 functional, respectively. Reprinted with permission from Ref. [35]. Copyright 2016 Royal Society of Chemistry. (c, d) The Ґ-X and Ґ-Y directions of dielectric function. Reprinted with permission from Ref. [52]. Copyright 2009 American Physical Society.

Figure 5(a) presents the band structure of borophene calculated with a settled Fermi level (EF), by the PBE functional in the direction of high symmetry of Brillion area, which is keeping with former theoretical work. The EF consists of three different bands, two follow the G-X direction and the other one follows the S-Y direction, along the directions indicating the metallic behaviors [35]. The crystal structure and the electronic band structure all reveal high anisotropy. The band structure displays metallic character while along one direction; nevertheless, a huge one is found while along the other direction [13]. Therefore, layered borophene behaves anisotropically in electronic properties rooting the anisotropic atomic structure [50].

It is important to obtain the exact electronic structure of borophene by hybrid functional calculations employing the HSE06 functional calculation. The band structure of borophene is shown in Figure 5(b). Compared with PBE functional calculation, the bandgap in HSE06 functional calculation increased by more than 11 eV, which was caused by the slight growth of conduction band minimum value (CBM) and the slight reduction of valence band maximum value (VBM) along the X-S direction [35].

Figures 5(c) and 5(d) show the optical absorption spectrum obtained by the imagined part of the dielectric function. It is found that two plasma branches exist in different energy ranges, that is, the high-energy branch (HE mode) and low-energy branch (LE mode), respectively. The HE mode is approximately linearly diffused and can extend to the ultraviolet region. However, the LE mode branch presents a relatively obvious anisotropic dispersion in different directions [51].

Because of the electron deficiency of elemental boron, it has all kinds of crystal structures including multicenter bonds. In contrast with the metallic, ionic, covalent, and van der Waals bonds, the multicenter bonds have extremely complex bonding type, which is vital because of its existence in abundant compounds [53]. The line defects in borophene have similar metallic structure to the original *v*1/6 and *v*1/5 sheets; thus, the electronic properties of borophene are relatively stable at room temperature for its underlying structural complexity. However, the delicate electronic modulations consistent with a CDW are prominent in the extremely low temperature [54, 55]. The chemical and electronic properties of borophenes are possible to be tuned by various chemical modifications. Consequently, the novel borophene and graphene may be the complementary partner [28].

### 2.3. Photoacoustic and Photothermal Properties

Usually, photoacoustic and photothermal signals are used for imaging-guided therapy [56–58]. In the very attractive area of nanomaterial-based cancer therapy, nanomaterials with distinct properties, for instance photothermal and photoacoustic transfer principles, have been deemed as an amusing and hopeful approach for the destruction of cancer cells [59, 60].

Kang et al. have announced the photoacoustic effect of small-sized material in an aqueous solution and studied the mechanism [61]. The intensity of photoacoustic amplitude produced a forceful shock wave and led to explosion like a firecracker at the nanoscale. The conversion from optical energy to acoustic energy could lead to a new discovery for using small-sized material as underlying therapeutic agents for cancer cell destruction [59].

For example, borophene can enter the cell at a very small size; the photoacoustic energy can be used both for cancer therapeutics and to generate acoustic waves on small-grained materials; it can lay a foundation for the application of efficient optical image generation in the future. The stress and pressure coming into being on the surface of nanomaterial during the photoacoustic therapy process can also be applied to photocontrolled release of anticancer drugs, iRNA, and proteins from the surface of small-sized material into the cells. Cancer is killed without giving rise to drug resistance and toxicity since such a photoacoustic process is a physical response that occurs in a short period of time. These new discoveries will be useful for the application of the photoacoustic properties and small-sized material structures in cancer therapeutic approaches [59].

The in vivo and in vitro results presented by Ji et al. proved the huge potential of the B-PEG (boron surface modification with polyethylene glycol) nanosheet for cancer photothermal chemotherapy [62]. They also developed the potential of the near-infrared light-induced hyperthermia of B-PEG nanosheet. The temperature of aqueous solution contains boron which was much higher than the pure aqueous solution under the same condition of NIR laser. The huge temperature variation further confirmed that our efforts are needed for preparation of the single-layer borophene. Because of its high photothermal conversion efficiency, boron nanosheet can be developed into effective materials for tumor treatment [62].

### 2.4. Metallic Properties

Metallicity is the most famous character of borophene in comparison with other semiconductors (e.g., phosphorene) or semimetals (e.g., silicene and graphene). Differing from bulk boron allotropes, borophene reveals metallicity which is in consistence with the anticipations of a greatly anisotropic 2D metal [25].

**Figure 6:**
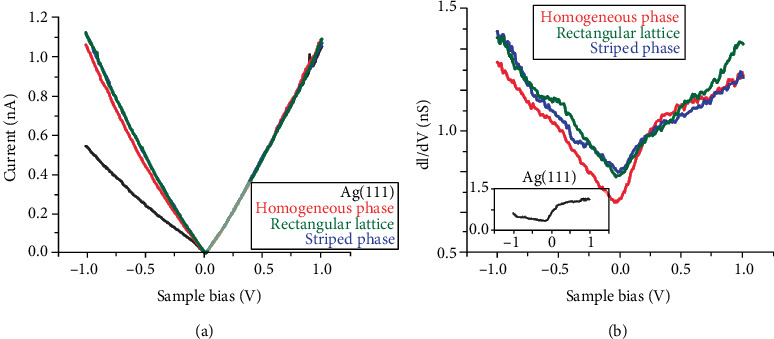
(a) The scanning tunneling spectroscopy (STS) current-voltage curves of borophene and (b) the scanning tunneling spectroscopy *dI*/*dV* spectra of borophene. Reprinted with permission from Ref. [25]. Copyright 2015 American Association for the Advancement of Science.

STS notarizes the metallicity of borophene through current-voltage curves, as shown in Figure 6(a), and the *dI*/*dV* spectra, as shown in Figure 6(b), which measure the DOS [25]. On the one hand, borophene is able to resist a large load, until the failure. On the other hand, the reactivity of borophene helps covalent bonding to the base that capacitates useful load transfer. Plenty of structural information on borophene also promoted researches on their electronic transport capacities. Meanwhile, if it holds a 2D structure, boron starts to display interesting metallic properties [46]. In particular, borophene could take along a high conductive electron density, *σ*, which does not depend on the Fermi level, in the near-visible range, opening a door to extend the plasmon energy [17].

**Figure 7:**
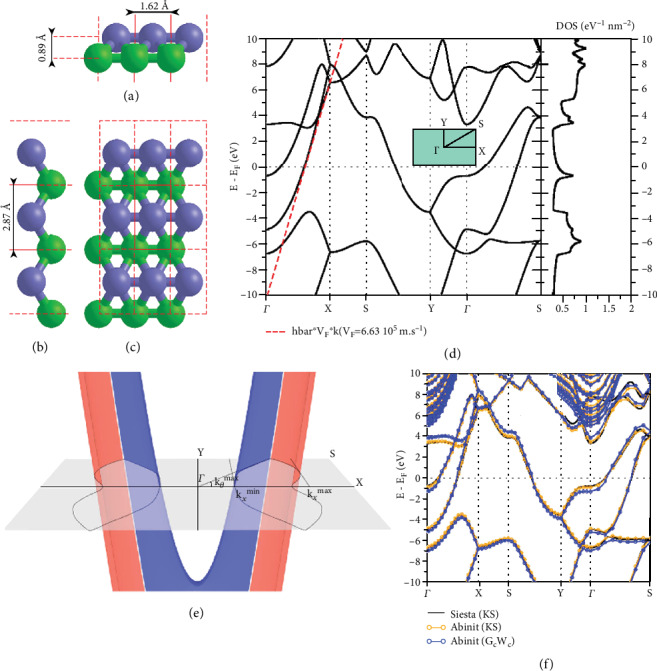
A 3 × 3 borophene super cell in (a, b) lateral views and (c) view from above. (d) DOS and electronic band structure with initial energy aligned to the EF. (e) 3D diagram of the two electronic energy bands crossing the EF in a TEW between −1ev and +1 eV. (f) The comparison between LDA with the G_0_W_0_ and KS band structures with Siesta GGA-PBE amended one. Reprinted with permission from Ref. [47]. Copyright 2016 IOP Publishing.

The lattice parameters of borophene are shown in Figures 7(a)–7(c), with the value of *a* = 1.6212 Å and *b* = 2.8699 Å. The value difference between bottom and top atoms can be up to 0.89 Å. Fermi velocity plays an important role in electrical conductivity, the electron-phonon relaxation time close to EF, and DOS at EF [47].  Figure 7(d) shows the electronic band structure, which reveals a priori and powerful anisotropic metallic feature as the bands are discovered to be deeply scattered in the *k*_*x*_ direction (*Γ*-X and Y-S), accompanied large group velocity at the value of 6.6∗10^5^ m per s.  Figure 7(e) reveals that electronic bands can often pass the Fermi level if a line is parallel to *k*_*x*_; however, it is different to the lines that were parallel to *k*_*y*_. Thus, the prediction was that transport may only happen till the choosy wave vectors *k* whose *k*_*x*_ constituent was in the permitted region. In the end, the recalculation and correction of the band structure were conducted by using the LDA method with ABI-NIT package and a one shot G_0_W_0_ approximation to the quasi particle problem. Kohn–Sham structure showed less different change to the G_0_W_0_ one. One can only realize an increase of the local bandgap at the *Γ* point and usually speaking an increase of the Fermi velocity [35, 47].

Mir et al. guess that due to the slight weight of boron, it may solidify electron-phonon combination; this may improve conventional, phonon-mediated superconductivity [17]. Boron reveals much more crossings through deeply dispersed (almost parabolic) bands. It indicates that compared to metallic MX_2_ compounds, 2D boron owns a huge free charge shipper concentration [63]. Therefore, borophene may be very useful as an electrode material in the future [54]. Moreover, after oxidation, borophene remains metallic and the oxidized borophene has an enormous improvement on both optical conductivity and optical property [47].

### 2.5. Semiconducting Properties

In recent years, due to the rapid development of the electronic industry, the traditional silicon technology can no longer meet the requirements of the semiconductors [64]. Hence, looking for a new semiconductor material to promote the development of the electronic industry is a crucial factor. Through research, Yang et al. found that borophene can form ideal contact with 2D semiconductor and effectively reduce the contact resistance, which can further improve the related performance of 2D transistor [34].

**Figure 8:**
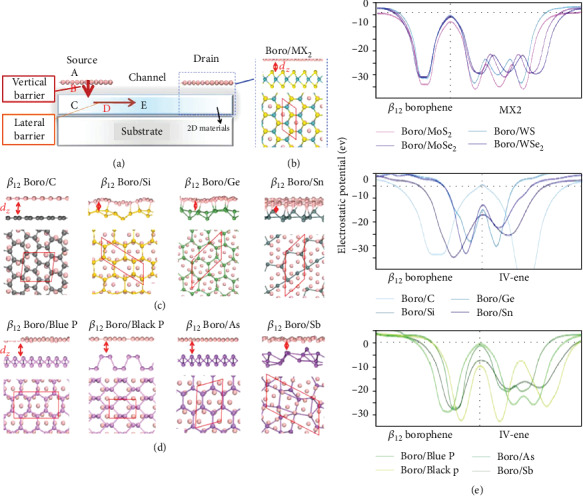
(a) *β*_12_ borophene is in contact with 2D semiconductor atoms. The electron injection monolayer *β*_12_ borophene shows its path (A ⟶ B ⟶ C ⟶ D ⟶ E) by the red arrow. Lateral and top viewports of the utmost steady structure: (b) *β*_12_ borophene gets to MX_2_ (M for Mo or W; X for S or Se); (c, d) *β*_12_ borophene gets to the group IV-enes. (e) The balanced electrostatic potential with *z* position for *β*_12_ borophene/2D material interactions. Reprinted with permission from Ref. [34]. Copyright 2017 Royal Society of Chemistry.

Jie et al. studied experimentally and systematically that the tunnel barrier was nearly zero after borophene making effective contact with various 2D semiconductors. It is shown in Figures 8(a)–8(d). The valid channel barrier height is given as the barrier height difference that must be overcome when the Fermi energy of the carrier in the metal is the same as that of the heterostructure. As shown in Figure 8(e), all of the tunnel barriers in the 2D layer in contact with borophene are zero, only graphene is 0.10 eV. Earlier findings have accorded that there is a tunnel barrier between the 2D material and bulk metal, so it can be seen that zero tunneling barrier is an irreplaceable favorable condition of borophene as a semiconductor contact layer.

The research on borophene is just beginning. With the development of the research on borophene, borophene not only has the above excellent properties but also may have novel atomic structure, excellent physical and chemical properties, and more interesting quantum effects, providing more possibilities for borophene-based applications in the future.

## 3. Experimental Fabrications

As is known to us all, a great challenge of fabricating borophene still exists due to the bonding configurations in bulk boron. Theoretically, a triangular lattice will be more stable if it has periodic holes [53, 65] and can grow on metal substrates, such as Ag(111) [66], Au(111) [67], and Cu(11) [68]. Owing to the metal passivation from stabilization of the sp^2^ hybridization and Al with the stabilization strategy of transferring one electron charge of Al to boron atom [69, 70], many researchers have synthesized atomically thin borophene via chemical vapor deposition (CVD), bottom-up fabrication techniques, top-down strategies, liquid-phase exfoliation, and sonochemical exfoliation techniques [25, 44, 62, 69, 71, 72]. These rare fabrication investigations show the difficult exfoliation from nonlayer bulk B and thus seriously limit the further application of borophene.

### 3.1. Bottom-Up Fabrication

#### 3.1.1. Physical Growth of Stripped Borophene

**Figure 9:**
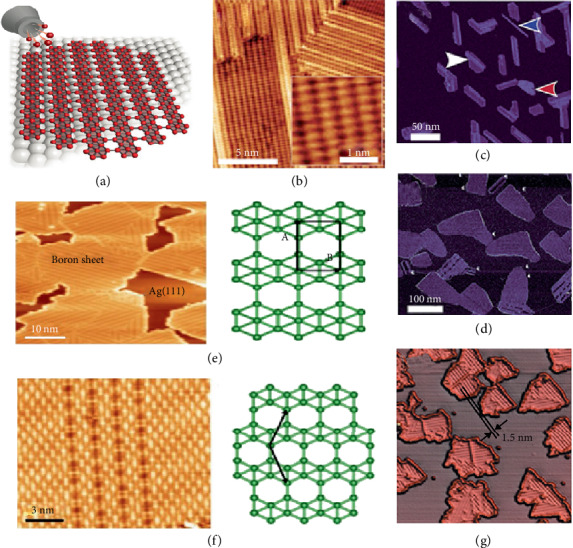
(a) Schematics of borophene growth. (b) STM topography pictures displaying striped-phase atomic-scale structure. (c) The sheets with striped phase and (d) homogeneous phase. Reprinted with permission from Ref. [25]. Copyright 2015 American Association for the Advancement of Science. (e) STM topography of *β*_12_ borophene, the atomic structure diagram of *β*_12_ borophene. (f) STM topography and atomic structure model of *χ*3 borophene. Reprinted with permission from Ref. [73]. Copyright 2019 American Chemical Society. (g) 3D STM topographic image. Reprinted with permission from Ref. [71]. Copyright 2016 Springer Nature Limited.

Mannix et al. firstly demonstrated the atomically thin borophene on the inert surface of Ag(111) with the morphology of striped-phase nanoribbon using molecular beam epitaxy (MBE) technique in Figures 9(a) and 9(b) [25]. Upon deposition at substrate temperature of 550°C, the morphology shows a striped pattern in Figure 9(c) and a homogeneous pattern in Figure 9(d). The deposition rate and substrate temperature control the growth of these two phases. Figures 9(c) and 9(d) indicate the stable state of striped phase and metastable homogeneous phase. A parallel fabrication work by Feng et al. shows that borophene grows on a Ag(111) substrate epitaxial [71]. Figure 9(e) reveals the STM of a surface which is covered with *β*_12_ borophene lumps. The atomic structure diagram of *β*_12_ borophene is rectangular [73]. Figure 9(f) reveals the STM topography and atomic structure model of *χ*3 borophene. The similar striped atomic arrangement can be obtained in both reported borophene in Figure 9(g).

Although the fabrication of borophene is successful, it is still unclear whether synthetic borophene can exist on structurally and chemically different layers. Boron's location in multiple chemical conditions is solved by sub-Angstrom spatial resolution, indicating that the borophene generates on planar layer which is larger than the Ag surface that is unreconstructed for about 2.4 A. It has the potential to develop wider diversity of 2D material through the separation from the growth substrate compared with the method of bulk layered crystal structures [74].

**Figure 10:**
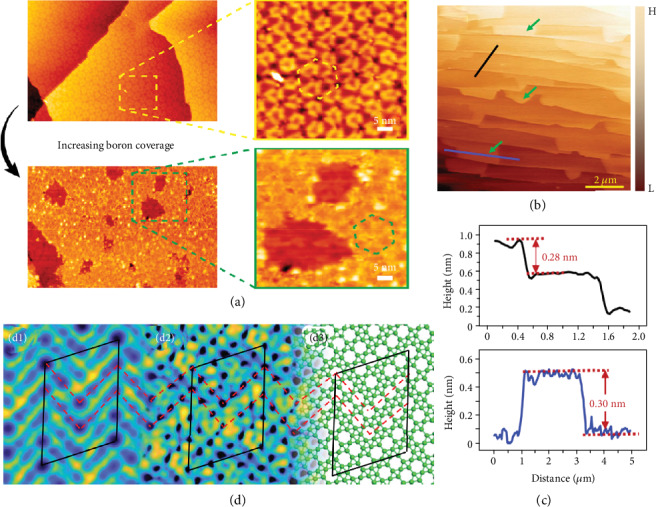
(a) Increasing boron dose leads to the breakdown of the network and growth of greater borophene islands. Reprinted with permission from Ref. [75]. Copyright 2019 American Chemical Society. (b) Topographic AFM image. (c) The line profile reveals a 2.8 Å tall atomic step of the Cu substrate (the black line in (b)). And the line profile reveals that the thickness of the borophene sheet in surrounding conditions is about 3.0 Å (the blue line in (b)). (d1) High-resolution STM data of borophene. (d2) DFT-imitated constant tunneling current isosurface of the proposed borophene structure. (d3) The diagram of the borophene structure with boron atoms and bonds shown in green. Reprinted with permission from Ref. [76]. Copyright 2019 Springer Nature Limited.

Different from the studied growth on Ag substrates, Kiraly et al. report that borophene islands can be generated under high temperatures with Au [75]. The method to produce borophene on Au(111) is different from the way that grows on Ag with only the surface (111). Importantly, the nucleation and growth of borophene are because of energy minimization and strain relief of the Au(111) surface. Owing to the increasing of boron coverage, the borophene changed from small well-organized islands to larger sheets, as shown in Figure 10(a). In Figures 10(b) and 10(c), the growth of one-atom thick boron islands has been proved in AFM.

The crystal structure is triangular with honeycomb lattice, and the exact ratio of honeycomb lattice sites and triangular sites is determined to be 1/5 in Figure 10(d). Furthermore, the first-principles calculations prove that the charge-transfer interaction occurs with minor degree of covalent bonding between borophene and Cu. This study opens a bright future for borophene-based device fabrication.

#### 3.1.2. Physical Growth of Honeycomb Borophene

Due to the planar honeycomb structure with sp2 hybridization in graphene, it makes it suitable for numerous promising applications [77], which leads to the research enthusiasm on other elemental 2D materials, for instance, silicene [78], germanene [79], and stanene [80]. However, these 2D materials are easy to form buckled honeycomb structure owing to the mixed sp2-sp3 hybridization as compared with sp2 hybridization in graphene with planar honeycomb structure. Fortunately, boron holds an even smaller atomic radius than carbon, rendering the possibility of forming planar honeycomb borophene.

Making the honeycomb borophene charming has three reasons. Firstly, in honeycomb lattice, the Dirac fermions have the similar electronic properties with group IV monoelemental-enes [81, 82]. Secondly, the honeycomb boron layer exists in MgB_2_ and it is just because of the honeycomb borophene that endows the high *T*_*c*_ superconduction [83]. Extraction of the honeycomb borophene would further optimize the superconductivity [49, 84]. Last but importantly, boron atom possesses three valence electrons, endowing the electron deficiency in honeycomb borophene and thus the unstable nature, which happens to satisfy the biodegradability need for biomedical applications.

The electron deficiency of boron makes honeycomb borophene challenging to be fabricated [69]. The honeycomb borophene can steadily exist in boride compounds (e.g., MgB_2_), which is because of the supplied electron for boron atoms from Mg atoms [85].

**Figure 11:**
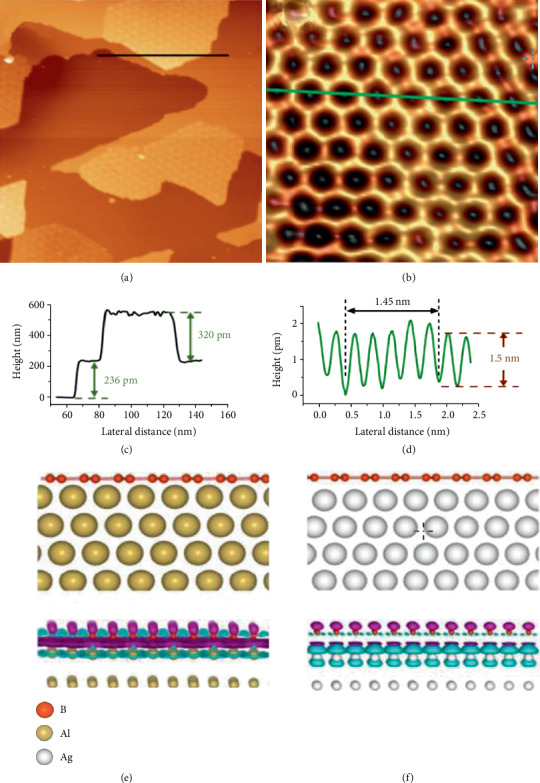
(a) STM image showing a one-atom thick boron island running across an Al(111) step. (c) Line profile corresponding to the black line in (a). (b) The STM image of boron. (d) Line profile corresponding to the green line in (b). (e) Side elevation of honeycomb borophene on Al(111). (f) Side elevation of honeycomb borophene on Ag(111). Reprinted with permission from Ref. [69]. Copyright 2018 Science China Press.

Li et al. successfully obtained the purely honeycomb, graphene-like borophene, through the way of using the Al(111) surface as the substrate; in the meantime, the molecular beam epitaxy (MBE) was also used which is grown in ultrahigh vacuum [69]. STM images apparently show the faultless monolayer borophene without buckled, planar honeycomb lattice alike as graphene in Figures 11(a) and 11(b). The line profile corresponding to the black line in Figure 11(a) is shown in Figure 11(c); the line profile corresponding to the green line in Figure 11(b) is shown in Figure 11(d). The honeycomb borophene on Al(111) is energetically firm which can be obtained by the theoretical calculations. It is worth noting that almost one electron charge is shifted to every boron atom from the borophene/Al(111) and steadies the honeycomb borophene structure; however, almost no charge was transferred in borophene/Ag(111) in Figures 11(e) and 11(f). The existence of honeycomb 2D allotrope can play an import role on the basic study of boron chemistry; it can also supply a meaningful platform with which boron-based materials can be fabricated.

Zhu et al. comprehensively studied the properties of honeycomb borophene, such as the structural and energetic properties, that held on Al(111) by calculations [70]. Their calculations show a fierce bond energy between the honeycomb borophene and the Al(111) substrate; compared with the research before which indicated the coactions between graphene and some transition-metal surfaces, it is enormously stronger. The fierce coactions can be illustrated through the charge and back donation and the solid covalent bonding coactions between the honeycomb borophene and the substrate. The strong bond interaction contributed a lot to the stabilization of honeycomb borophene.

#### 3.1.3. CVD Growth

**Figure 12:**
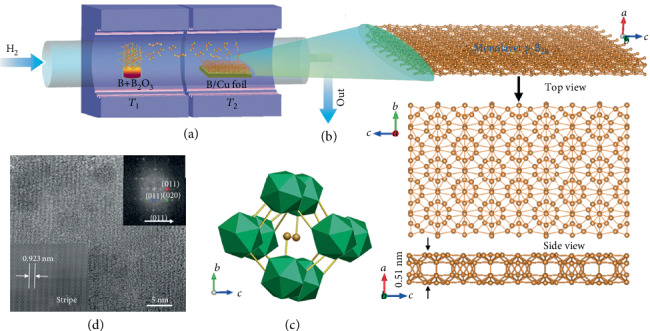
(a) Diagram of the homemade two-zone CVD furnace for growing borophene. (b) Top and lateral views of the borophene. (c) Atomic space structure of the elementary cell for borophene. (d) The striped phase shown by HRTEM image. Reprinted with permission from Ref. [44]. Copyright 2015 John Wiley & Sons, Inc.

Besides the physical growth, Tai et al. synthesized the borophene on Cu foils by chemical vapor deposition (CVD), as shown in Figure 12(a) [44]. Through elaborately designing the CVD furnace, the condition of the source region (T1) and the growth substrate region (T2) can be independently manipulated. The temperature of the T1 zone was set to be 1100°C to obtain the growth vapor, and the temperature of the T2 zone was set to be 1000°C to anneal the Cu foil. The borophene consists of orthorhombic g-B28 cells, which include B2 dumbbells and icosahedral B12 components in Figures 12(b) and 12(c). Although the growth techniques are totally different from the physical vapor epitaxy, the striped phase is also observed in Figure 12(d).

### 3.2. Top-Down Fabrication

**Figure 13:**
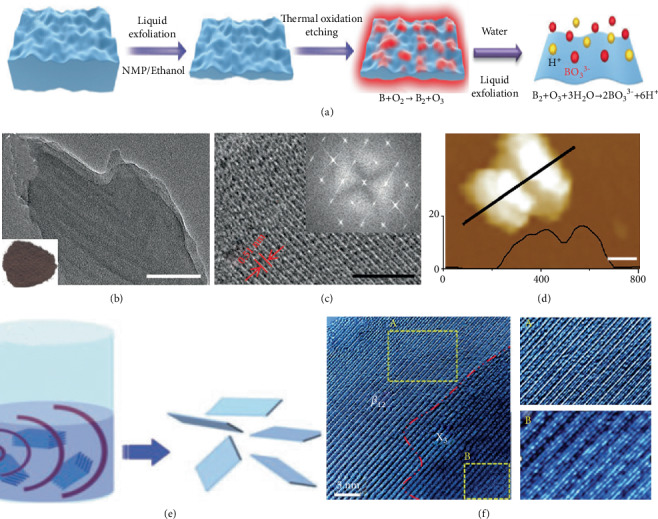
(a) Schematic representation of borophene. (b) HRTEM image, (c) TEM image, and (d) AFM image. Reprinted with permission from Ref. [62]. Copyright 2018 John Wiley & Sons, Inc. (e) Diagrammatic drawing of liquid-phase exfoliation preparation of borophene. (f) HRTEM of borophene sheet with two phases. Reprinted with permission from Ref. [86]. Copyright 2018 Royal Society of Chemistry.

The research on another efficient fabrication technique, i.e., direct exfoliation, is rarely reported because boron exhibits complex bonding network, rendering the direct exfoliation challenging. Ji et al. synthesized the borophene through a top-down approach, which can be divided into two times of liquid exfoliation and one thermal oxidation etching in Figure 13(a) [62]. After the first exfoliation, the B nanosheets appear to be thick. Then, the thick nanosheets can be oxidized to B_2_O_3_, which further dissolves into water. Later, the second ultrasonic exfoliation was employed. Through these three steps, the B nanosheets with lateral size limit 100 nm and thickness less than 5 nm can be obtained (Figures 13(b)–13(d)). This fabrication strategy is fit for biomedical applications.

An easy and massive synthesis of atomic sheets of borophene through a fresh liquid-phase exfoliation and the reduction of borophene oxide is proved by Jiang et al. in Figure 13(e) [86]. Electron microscopy verified the existence of *β*_12_, *χ*3, and their intermediate phases of borophene in Figure 13(f). These borophene materials and their hybrids will create great contributions in the realm of 2D materials and could contribute to develop future generations of apparatus and emerging applications.

The honeycomb borophene layer existing in diborides may also provide an avenue for obtaining honeycomb borophene directly through the top-down fabrication strategy [87, 88]. However, the sandwiched boron layer is strongly bound with two external metal layers through covalent bond, endowing the direct exfoliation of honeycomb borophene a big challenge.

## 4. Applications

### 4.1. Energy Applications

Larger capacitance, good electrical conductivity, and ionic conductivity are the key to whether a material can be used as electrode material [89]. As the 2D material, borophene has high surface liveness, which is conducive to the realization of super high storage capacity of metal material. In addition, the metallic band construction of borophene facilitates the conduction of electricity, so it can also be used as the electrode for metal material ion batteries.

**Figure 14:**
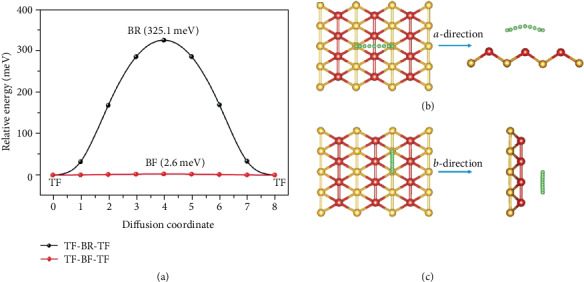
(a) Energy barriers and (b, c) pathways of Li movement alongside serrate and armchair directions. Reproduced from Ref. [90]. Copyright 2016 Elsevier B.V.

Because of the high surface activity of borophene, borophene as an electrode material has a strong interaction with lithium ion. The initial intercalation voltage is 1.12 eV, which reduces with the increase of Li adsorption range. Jiang et al. first proposed sodium borohydride as an excellent cathode material for lithium batteries [90]. The fully lithiated phase of 2-Pmmn is much higher than traditional graphite [90], silicene [91], phosphorene [92], and other electrode materials. As shown in Figure 14, the relative migration potential barrier of lithium ions in borophene is significantly smaller than that in graphite [90], silicene [92], phosphorene [91], and Li_4_Ti_5_O_12_, which is only 2.6 meV. The longitudinal potential barrier of borophene is about 325 meV, so the lithium ions on its surface have strong anisotropy during the migration process, and during the whole lithium process, the electronic structure of the lithium is characterized by the same characteristics of metal elements, indicating that borophene has good conductivity. To sum up, because borophene has high ionic conductivity and excellent electronic conductivity, it has relatively excellent performance in the whole charging and discharging process.

On the other hand, we can adjust properties of materials through doping, so as to realize the complementary advantages between materials [93]. Borophene hydride has a volume of 504 mAh/g; borophene has a volume of 1860 mAh/g; as a result, scientists in the study of borophene olefin as the anode of lithium-ion batteries when joined by hydrogen, due to the charge in the process of transfer, take borophene transfer to the hydrogen atom, so the borophene phase increases, thus reducing the interaction between lithium and borophene hydride. At the same time, the application of borophene in phases 12 and 3 in sodium and magnesium ion batteries also showed excellent characteristics. Lithium batteries using borophene in phases 12 and 3 as anode material were 1984 mAh/g and 1240 mAh/g, which were far beyond traditional graphene, silicene, phosphorene, and Li_4_Ti_5_O_12_. The migration energy potential barrier of sodium borate ion in stage 12 is 330 meV, which is lower than that of lithium ion (660 meV). The migration energy barrier of sodium borate at phase 3 was much lower than that of lithium at 660 meV. It can be seen from these reference data that borophene containing borophene vacancy is used as the cathode material of sodium ion battery, which has higher performance than traditional lithium ion battery in all aspects.

To sum up, theoretically, borophene has a high capacity, excellent conductivity, and efficient ion transport capacity, providing a broader prospect for the development of cathode materials for lithium, sodium, and magnesium ion batteries [94–98].

In recent years, the development of lithium-sulfur batteries has been seriously hindered; however, the right sulfur anchoring material can inhibit the shuttle effect [99]. The interaction between the lithium polysulfide and the anchoring material should be just right. As shown in Table 1, for graphene, the adsorption energies are 0.65 eV and 0.72 eV, respectively, for Li_2_S_4_, Li_2_S_6_, and Li_2_S_8_. Because of such interactions, graphene cannot be used as an anchoring material for polysulfide.

Zhao et al. have explored the application of borophene benzene as a potential anchoring material for lithium-sulfur batteries, using a first-principles calculation method [86, 103, 104]. The adsorption energies on the 2-Pmmn phase of borophene benzene are 6.45, 4.32, and 6.18 eV, respectively, for Li_2_S_4_, Li_2_S_6_, and Li_2_S_8_. Lithium polysulfide will be decomposed, and irreversible sulfur loss will occur due to interaction during absorbing and releasing electrical energy. Surprisingly, the mutual effect between lithium polysulfide and borophene benzene *χ*3 phase is much smaller than that between lithium polysulfide and borophene benzene 2-Pmmn phase. The energies of lithium Li_2_S_4_, lithium Li_2_S_6_, and lithium Li_2_S_8_ on the borophene benzene *χ*3 phase are 2.67, 2.53, and 2.87 eV, respectively, indicating that the borophene benzene *χ*3 phase is a perfect fixing material and can be used in lithium sulfur batteries. During the charging and discharging process of the battery, the appropriate adsorption strength is beneficial to inhibit the adsorption of spindle carbon on the electrode and protect its cycling structure from decomposition. The three phases of the borophene are a useful grappling material for batteries because of the metal structure characteristic of the borophene during the whole battery charging and discharging cycle. On the other hand, the borophene in phase 12 also showed good feature as a fixing material for lithium batteries. Moreover, the borophene of the *β*_12_ also exhibits metallic properties throughout the battery cycle. Thus, *χ*3 and *β*_12_ are promising anchoring materials for lithium batteries.

### 4.2. Optoelectronic Applications

#### 4.2.1. Sensor Application

*(1) Gas Sensor*. Conventional carbon nanotubes and graphene gas sensors are highly sensitive to several irritating gases, but highly toxic chemicals such as formaldehyde cannot be identified. In recent years, the wide application prospect of borophene in gas sensors has attracted extensive attention [101].

In recent years, based on the DFT, researchers have studied the application prospect of borophene nanometer as formaldehyde sensor [101]. Through calculation and reasoning analysis, when HCOH molecule exists, the conductivity of borophene increases significantly, thus generating electrical signals. With the adsorption of more formaldehyde molecules by borophene, the strength of electrical signals increases, indicating that the sensitivity of borophene to formaldehyde gas is relatively high. In addition, borophene has high surface volume ratios and can be used to detect low-concentration gas molecules [102].

**Figure 15:**
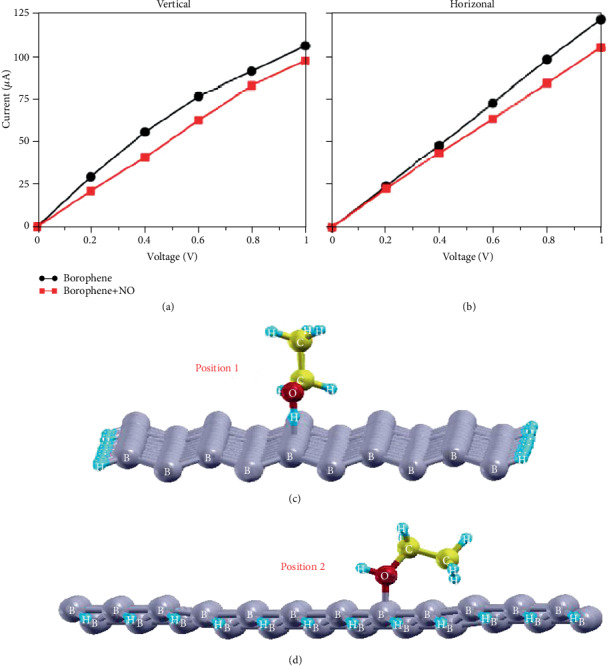
Current-voltage characteristics of borophene with and without adsorption of NO. The current flows in (a) vertical and (b) horizontal. Reprinted with permission from Ref. [101]. Copyright 2018 Journal of Physical Chemistry C. (c) Adsorption of ethanol molecules on position 1 and position 2. Reprinted with permission from Ref. [102]. Copyright 2017 Elsevier B.V.

Huang et al. which studied in recent years have been able to succeed with bending and linear defects of preparation phase of the 2D borophene command on the NO gas molecular adsorption ability, by bending test and linear borophene in the adsorption NO gas after the *i*-*v* characteristic curve, as shown in Figures 15(a) and 15(b); we can clearly see the sensitivity of the borophene to NO on electrical properties [102].

In addition, the borophene nanosheet also adsorbs ethanol. The adsorption of nanostructure borophene on oxygen atoms and hydrogen atoms on ethanol molecules is shown in Figures 15(c) and 15(d), which is at positions 1 and 2, respectively. Due to the adsorption of borophene and oxygen atoms and hydrogen atoms on ethanol, the bandgap of nanoborophene decreases rapidly and the conductivity increases. Therefore, the changes of adsorption energy and bandgap can be used to detect ethanol vapor.

#### 4.2.2. The Electrocatalytic Applications

In practical applications, ideal gas adsorbents cannot meet the test requirements because of their weak adsorbability when adsorbing gas, nor can they affect the release after adsorption because of their strong adsorbability. Therefore, it is a great challenge to choose a material with appropriate adsorbability and good selectivity.

By using density functional theory (DFT) calculation method, extra electrons to the adsorbent are added. Tan et al. show that the borophene with negative charge on CO_2_ adsorption is more than before; in the experimental condition to achieve CO_2_ saturation coverage, adsorption amount has reached 6.73 × 10^−14^ cm to 2 × 10^−14^ cm [105, 106].

**Figure 16:**
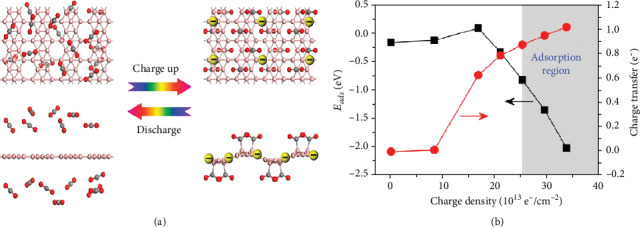
(a) CO_2_ molecules adsorbed negatively charged borophene more strongly after the addition of extra electrons to the adsorbent. (b) CO_2_ adsorption energy of borophene with extra electrons at B_3_ site and the charge transfer from borophene to CO_2_ molecule as functions of charge densities. The adsorption region is represented by the gray region. Reprinted with permission from Ref. [105]. Copyright 2017 American Chemical Society.

On the other hand, other materials cannot release CO_2_ effectively after absorbing it, while the process of absorbing and releasing CO_2_ of negatively charged borophene is reversible, because we can control the absorption and release of borophene by controlling whether it is negatively charged, as shown in Figure 16(a), so as to realize electrical catalytic function.

As shown in Figure 16(b), the sensitivity of CO_2_ to charge density in the region of small negative charge density is much less than that in the region of large negative charge density. And adsorption on carbon and borophene in borophene associated with negative charge, when the borophene suggests that the negative charge increase, will transfer to CO_2_, so borophene above negative charge with CO_2_ above the repellency between the negative charges will also increase; therefore, in a small negative charge density of the area, more is absorbed.

### 4.3. Biological Applications of Borophene

#### 4.3.1. Biomedical Applications

In recent years, PA imaging is a particularly promising biotechnology biophoton radiation diagnosis method because of its advantages of deep inspection, depth resolution 3D shadow, sensitive dexterity, stereo resolution, and image sharpness [107–109]. Because B-PEG NSs prepared by 2D borophene showed excellent photothermal performance, Ji et al. conducted in-depth research on the prospective of B-PEG NSs as PA carrier in mice [62].

**Figure 17:**
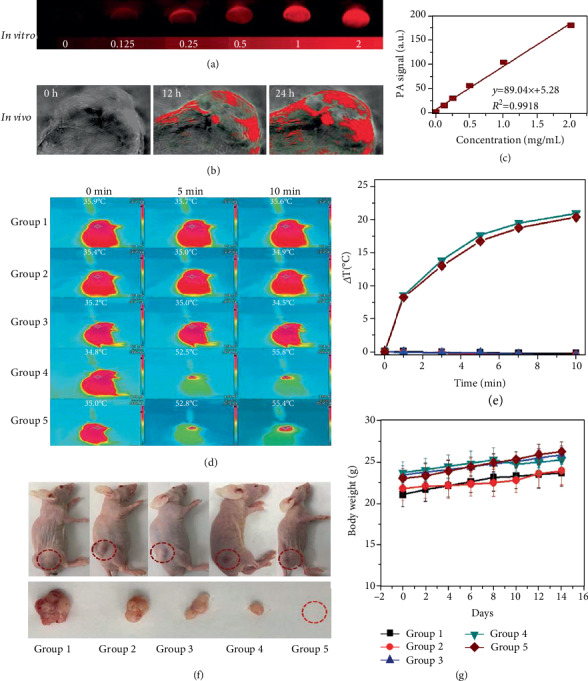
(a) In vitro PA pictures of using B-PEG NSs. (b) PA imaging of tumor sites over time (1, 12, and 24 h) post injection. (c) PA values of using B-PEG NSs. (d) Infrared imaging. (e) Temperature changes with time in the MCF7 tumor-bearing mice after different handlings. (f) The tumor sites of each group were photographed after 14 days of treatment. (g) Changes in body weight were recorded during the experiment. Reprinted with permission from Ref. [62]. Copyright 2018 Wiley-Blackwell.

Because of its excellent properties in depth resolution 3D shadow, sensitive dexterity, stereo resolution, and image sharpness, PA shadow has become one of the most promising biophotonic radiation diagnosis methods. Because of the excellent photothermal properties of B-PEG NSs in this study, the possibility of using B-PEG NSs as PA carrier in mice was experimentally investigated. As observed in Figure 17(a) with the increase of B-PEG NSs concentration, PA signal also becomes stronger and stronger. On the other hand, it can also be seen from Figure 17(c) that there is a good linear relationship between PA signal and B-PEG NSs concentration, so it can be inferred that B-PEG NSs is a good PA reagent. And as time went on, it was found that the PA signal became stronger and stronger, it is shown in Figure 17(b).

As shown in Figures 17(d) and 17(e), the changes in body temperature of mice in different treatment groups were recorded by infrared thermography, and it was found that the body temperature of mice in the group receiving near-infrared irradiation was significantly lower than that in the group without exposure. In addition, a large number of statistics and experiments have found that mice treated with chemotherapy-photothermal combination therapy showed extremely high tumor growth inhibition, indicating the good therapeutic effect of B-PEG/DOX NSs. As shown in Figure 17(f), the volume of tumor generated in mice can be clearly seen by comparison. The treatment has not found disturbing side effects such as abnormal weight loss (Figure 17(g)), eating, drinking, activity, or neurological problems.

#### 4.3.2. Biosensor

In the field of biomedicine, borophene as a nanomaterial has been widely studied and paid attention to because of its good flexibility. This is because borophene has excellent molecular, physical, and mechanical properties in strip and tubular conformations.

In the biomedical applications of nanomaterials, borophene is often used as a 2D nanomaterial combined with other metals and semiconductor materials to improve the performance of biological applications without any negative effects [28]. DNA sequencing has been realized by different methods through the determination of base sequence, which has a great impact on the decoding of human biological code [110–112]. However, the application of nanomaterials in this technology will greatly accelerate the research of biology and medicine [82, 113–118].

Borophene is effectively used for biosensors. By testing the energy and electron sensitivity of 2D borophene to the four bases (adenine (A), guanine (G), thymine (T), and cytosine (C)) on top of biological DNA, the scientists found that different bases attached to borophene produce different conductivities, resulting in different electrical signals. In addition, Das et al. also measured the sensitivity of borophene to different bases, A > G > C > T [119]. Thus, borophene nanosheets could be used in biological DNA sequencing devices.

**Figure 18:**
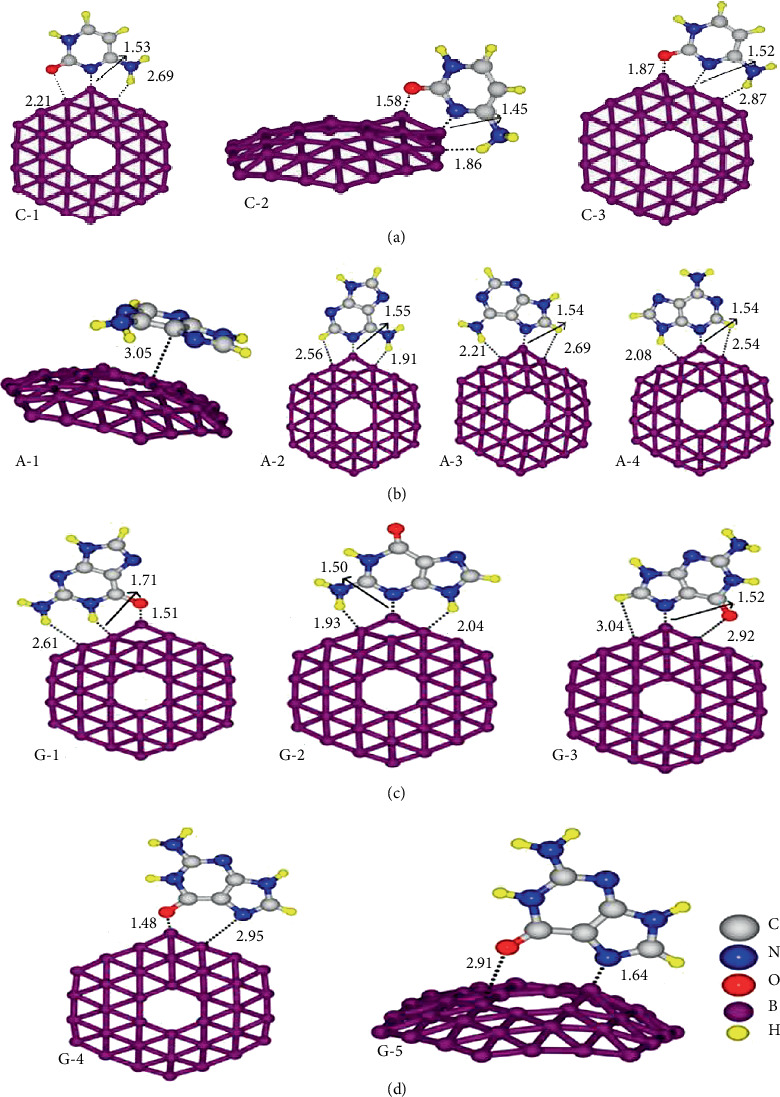
(a) Different adenines combine with borophene. Distances are in Å. (b) Different adenines combine with borophene. Distances are in Å. (c, d) Different adenines combine with borophene. Distances are in Å. Reprinted with permission from Ref. [120]. Copyright 2017 Elsevier.

Through the test and calculation, Rastgou et al. found that because the electronic sensitivity of borophene in different bases is different, the conductivity of borophene alkene will be different because of the action of different bases, as shown in Figure 18. In addition, the energy sensitivity of borophene alkene to different bases is also different, so the borophene alkene will have a broad application prospect in biological DNA sequencing [120].

Borophene looks more exciting than graphene. The “next generation of super nanomaterials” may be on the way. The borophene is expected to have a broader application and revolutionize energy, sensors, catalysts, and many other fields.

## 5. Challenge and Perspectives

This review systematically summarizes and classifies the recent developments of the borophene in terms of fabrications, properties, and various applications. We also provide our vital insights in the next discussions, aiming at placing the emerging borophene in the pole position for energy, sensor, and biomedical applications.

The iterative feedback loop between theoretical and experimental results is widely considered to be the key to expediting the discovery and exploitation of new materials with desired properties. On the basis of theoretical research, the successful synthesis of borophene, along with the generation of other 2D crystal materials which have no layered bulk form, introduces a new way for the production of 2D materials by manipulating the intricate chemistry of materials. The great consistency between theory and experimental practice for borophene indicates that further predictions are needed for future experimental investigations.

In the energy-related application of borophene, the good metal properties endow it to be a cathode material for extraordinary energy density in lithium batteries. Borophene also shows great potential in the application of biosensors. Borophene has different electrical sensitivities to different nucleobases, and it has been developed into a promising application in the sequencing of DNA of biological genetic material, which may be a key step for humans to crack biological genetic code. In addition, when borophene is in contact with gas, its resistance varies greatly, which is promising for gas sensors. In terms of biological application, borophene has excellent biocompatibility but its NIR absorption needs to be further enhanced.

Although the properties and synthesis methods of borophene have been widely studied, the applications are still less. Moreover, more theoretical work has been conducted but the experimental work is still.

At present, the synthesis of borophene suffers from low yield, and the borophene nanosheets are limited to be small sizes. In the future, the researchers need to improve the synthesis methods to achieve the large area and high-quality borophene nanosheets. Whether all-boron electronics could be realized in a manner similar to all-carbon electronics should be explored. Borophene applications can be extended from electronics to photovoltaic applications owing to its metallic nature. Most importantly, the low mechanical strength of borophene indicates its promising potential on wearable devices. For biomedical application, the Boron Neutron Capture Therapy (BNCT) can be further exploited by employing 2D borophene.

## Figures and Tables

**Table 1:** The adsorption energies (in eV) of Li_2_S, Li_2_S_2_, Li_2_S_4_, Li_2_S_6_, and Li_2_S_8_ on 2-Pmmn, *χ*3, *β*_12_ borophene, graphene, and phosphorene [86, 100]. Reproduced from Ref. [99]. Copyright 2019 Springer Science.

Species	Li_2_S	Li_2_S_2_	Li_2_S_4_	Li_2_S_6_	Li_2_S_8_	Ref.
2-Pmmn			6.45	4.32	6.18	[90]
*χ*3			2.67	2.53	2.87	[90]
*β*_12_	3.34	2.89	1.45	1.53	1.36	[101]
Graphene			0.65	0.72	0.73	[102]
Phosphorene	2.51	1.91	1.27	1.00	1.12	[102]
